# Changes of quality of life and cognitive function in individuals with Internet gaming disorder: A 6-month follow-up

**DOI:** 10.1097/MD.0000000000005695

**Published:** 2016-12-16

**Authors:** Jae-A Lim, Jun-Young Lee, Hee Yeon Jung, Bo Kyung Sohn, Sam-Wook Choi, Yeon Jin Kim, Dai-Jin Kim, Jung-Seok Choi

**Affiliations:** a Department of Psychiatry, SMG-SNU Boramae Medical Center; b Department of Psychiatry, Seoul National University College of Medicine; c Korea Institute on Behavioral Addictions, True Mind Mental Health Clinic, Seoul; d Korea Health Care and Information Research Institute, Namseoul University, Cheonan; e Department of Psychiatry, Seoul St Mary's Hospital, The Catholic University of Korea College of Medicine, Seoul, Republic of Korea.

**Keywords:** inhibitory control, Internet gaming disorder, psychological well-being, quality of life, response inhibition

## Abstract

Internet gaming disorder (IGD) contributes to poor quality of life (QOL) and cognitive dysfunction and is increasingly recognized as a social problem in various countries. However, no evidence exists to determine whether QOL and cognitive dysfunction stabilize after appropriate management. The present study addressed improvement in QOL and cognitive functioning associated with changes in addiction symptoms following outpatient management for IGD. A total of 84 young males (IGD group: N = 44, mean age: 19.159 ± 5.216 years; healthy control group: N = 40, mean age: 21.375 ± 6.307 years) participated in this study. We administered self-report questionnaires at baseline to assess clinical and psychological characteristics, and conducted traditional and computerized neuropsychological tests. Nineteen patients with IGD completed follow-up tests in the same manner after 6 months of outpatient treatment, which included pharmacotherapy with selective serotonin reuptake inhibitors. A baseline comparison of patients with IGD against the healthy control group showed that the IGD patients had more symptoms of depression and anxiety, higher degrees of impulsiveness and anger/aggression, higher levels of distress, poorer QOL, and impaired response inhibition. After 6 months of treatment, patients with IGD showed significant improvements in the severity of IGD, as well as in QOL, response inhibition, and executive functioning. Additionally, a stepwise multiple regression analysis revealed a favorable prognosis for IGD patients with low working memory functioning and high executive functioning at baseline. These results provide evidence regarding longitudinal changes in QOL and cognitive function following psychiatric intervention for IGD. Furthermore, it appears that response inhibition may be an objective state marker underlying the pathophysiology of IGD.

## 1 Introduction

Internet gaming disorder (IGD) is defined as an excessive and prolonged Internet gaming pattern that causes cognitive and behavioral problems and is generally conceptualized as a behavioral addiction that includes core components of addictive behaviors, progressive loss of control over gaming, tolerance, and withdrawal symptoms.^[1–3]^ IGD shares core clinical features with gambling disorder as a behavioral addiction cluster.^[4,5]^ The cognitive behavioral model of IGD suggests that individuals who suffer from psychosocial problems are more likely to develop IGD.^[6,7]^ IGD is associated with stress,^[8–11]^ aggressiveness, reduced work or academic achievement, and reduced well-being.^[2,3,12–14]^ Carli et al^[15]^ provided a systematic review of IGD and psychopathology and reported significant associations between IGD and depression, anxiety, symptoms of attention deficit hyperactivity disorder, obsessive-compulsive symptoms, and hostility/aggression. Furthermore, psychological and pharmacological interventions are “highly effective” for reducing IGD symptoms and comorbid depression and anxiety.^[16,17]^

Quality of life (QOL) is associated with a stronger sense of community and better emotional, physical, and material well-being.^[18]^ Whang et al^[19]^ reported that patients with IGD show high levels of loneliness, depression, and compulsiveness relative to nonaddicts, who have relatively low levels of loneliness, depression, and compulsiveness; these results suggest better psychological well-being among nonaddicts. In addition, 1 study indicated that patients with IGD have poor QOL relative to that of healthy controls (HCs).^[20]^ Ratings of overall QOL and subjective well-being decrease significantly relative to controls among other populations of addicts, including those with alcohol, gambling, or drug addictions.^[18,21]^ However, although IGD has received a great deal of attention from researchers, few studies have evaluated patients with IGD in terms of their QOL or psychological well-being. In addition, most studies investigating QOL of patients with IGD have adopted a cross-sectional design. To address the existing gaps in the literature, we conducted a longitudinal study of subjective QOL at baseline and after 6 months of treatment in patients with IGD to observed changes in subjective QOL and the association between QOL and changes in IGD symptoms following treatment. We expected that patients with IGD would show increased QOL after treatment based on a study indicating that treatment of substance use disorder (SUD) improves QOL at follow-up.^[22]^

In addition, our research addressed cognitive dysfunction in patients with IGD. A growing number of neurocognitive and neuroimaging studies have begun to investigate the neurobiological basis of IGD and, in particular, the changes in brain activity associated with impulsivity, compulsivity, and sensitivity to reward and punishment.^[23]^ Lee et al^[24]^ compared the degree of trait impulsivity evident in a group of patients with IGD against a group of pathological gamblers and suggested that IGD can be conceptualized as an impulse control disorder and that trait impulsivity is a marker of vulnerability to IGD. Similarly, Choi et al^[25]^ reported impaired inhibitory control as a core neurocognitive factor in patients with IGD. It is notable that impaired self-control (impaired inhibitory and executive control) has been established as a hallmark of addictive behaviors and impulse-control disorders.^[3,26]^ Impaired executive control seems to be a crucial factor with regard to IGD.^[3,9]^ Furthermore, problems with executive control have been well established in research pertaining to SUD. Disruptions in inhibitory or executive control are relevant to maintaining drug use behavior and the difficulty many individuals have resisting habitual drug use once this pattern of behavior has been established.^[27–31]^ Taken together, IGD has multiple features in common with drug addiction, including enhanced impulsivity, cognitive inflexibility, and attentional biases. However, it is not yet known whether cognitive dysfunction represents a pre-existing factor predisposing individuals to IGD or whether it develops as a result of excessive Internet gaming, or is a combination of both.^[9]^

In the present study, we investigated changes in QOL and cognitive functioning following outpatient management of patients suffering from IGD, and their relationship to changes in IGD symptoms. In addition, we examined predictive markers of symptom improvement following outpatient management. No study has evaluated before- and after-treatment effects in patients with IGD to offer predictions regarding the treatment prognosis for patients with IGD. Based on previous reviews of IGD and related addiction treatment studies,^[22,31]^ we hypothesized that patients with IGD would show improved executive functioning, particularly inhibitory control ability, and that they would report increased subjective QOL and psychological well-being after 6 months of outpatient management.

## 2 Methods

### 2.1 Study participants

A total of 84 young males participated in this study: 44 males were diagnosed with IGD (age: 19.159 ± 5.216 years) and 40 males were HCs (age: 21.375 ± 6.307 years). All patients had sought treatment at the outpatient clinic of SMG-SNU Boramae Medical Center in Seoul, South Korea, due to excessive Internet gaming.

Patients with IGD were diagnosed by an experienced psychiatrist, according to Diagnostic and Statistical Manual of Mental Disorders, 5th Edition criteria; Young Internet Addiction Test (IAT)^[32]^ was used to assess IGD severity. We only included patients with IAT scores ≥70 who spent more than 4 h/d and 30 h/wk using Internet games, with the aim of studying only those with severe IGD rather than those at high risk for developing the disorder due to excessive Internet gaming. In addition, the Structured Clinical Interview for DSM-IV was used to identify past and current psychiatric illnesses. Following completion of the clinical assessments and neuropsychological tests, 19 of the 44 patients completed 6 months of outpatient management including pharmacotherapy with serotonin reuptake inhibitors. The 19 patients with IGD underwent follow-up clinical assessments and neuropsychological tests. The primary treatment outcome was a change in the pre- to post-treatment IAT score.

The HCs were recruited from the local community and had no history of any psychiatric disorder. The HCs played Internet games <2 h/d. Exclusion criteria were a history of significant head injury, seizure disorder, mental retardation, or psychotic disorder. All participants were medication-naive at the time of assessment. The short form of the Korean-Wechsler Adult Intelligence Scale (K-WAIS SF) was administered to all subjects at baseline to estimate their intelligence quotient (IQ).

The Institutional Review Board of the SMG-SNU Boramae Medical Center approved the study protocol (IRB No 20120529/06-2012-119/120), and all subjects provided written informed consent prior to participation.

### 2.2 Measures

#### 2.2.1 Young Internet Addiction test

This instrument is composed of 20 items rated on a 5-point scale, where 1 indicates “very rarely” and 5 indicates “very frequently.” Total scores were calculated according to Young method, with a possible score for all 20 items of 20 to 100. Those who scored 20 to 39 were classified as “average users,” those who scored 40 to 69 were classified as “experiencing frequent problems,” and those who scored 70 to 100 were classified as suffering from “significant problems” due to Internet use.^[25,32]^ We used a version of the IAT that was validated for Korea.^[33]^

#### 2.2.2 Beck Anxiety Inventory

The Korean version of the Beck Anxiety Inventory (BAI)^[34,35]^ consists of 21 symptoms rated on a 4-point scale and measures the severity of certain symptoms experienced during the past week. Scores for all 21 items are summed to yield a single anxiety score.^[25]^ A total score of 0 to 7 indicates a minimal level of anxiety, 8 to 15 indicates mild anxiety, 16 to 25 indicates moderate anxiety, and 26 to 63 indicates severe anxiety.^[36]^

#### 2.2.3 Beck Depression Inventory

The Korean version of the Beck Depression Inventory (BDI)^[37,38]^ is a 21-item self-report questionnaire in which each item consists of 4 statements reflecting different levels of severity of a particular symptom experienced during the past week. Scores for all 21 items are summed to yield a single depression score.^[25]^ A total score of 0 to 13 is classified as reflecting minimal depression, 14 to 19 as mild depression, 20 to 28 as moderate depression, and 29 to 63 as severe depression.^[37]^

#### 2.2.4 Psychosocial Well-Being Index

Level of distress was measured using the 45-item Psychosocial Well-Being Index (PWI).^[39]^ This tool was developed based on the General Health Questionnaire created by Goldberg,^[40]^ which was designed to evaluate the psychological stability of community members and was subsequently adapted to address characteristics of the Korean population. It contains questions about the participant's physical and psychological status during the last few weeks, including performance of social roles, self-confidence, depression, sleep disturbances, anxiety, and general well-being. Higher scores on the PWI reflect higher levels of distress.^[20]^

#### 2.2.5 Barratt Impulsiveness Scale-11

The Barratt Impulsiveness Scale-version 11 (BIS-11)^[41,42]^ assesses impulsivity using a 4-point scale in which 1 indicates “rarely/never” and 4 indicates “almost always/always.” This instrument has yielded positive correlations with neuropsychological measures of impulsivity and is sensitive to executive function deficits in prefrontal and orbitofrontal systems in multiple clinical samples.^[43,44]^ For example, this instrument can significantly discriminate between problem gamblers and nongamblers, with problem gamblers tending to have higher scores.^[45,46]^

#### 2.2.6 State-Trait Anger Expression Inventory

The State-Trait Anger Expression Inventory (STAXI)^[47,48]^ assesses how a person feels at a given moment (state anger; e.g., “I am mad”) and how frequently and intensely the person feels angry (trait anger; e.g., “I get angry when slowed down”). Items are rated on a 4-point scale with 1 indicating “not at all” and 4 indicating “almost always.”^[49,50]^ This inventory provides separate measures for the experience and expression of anger; however, we were interested only in the expressive domain of anger; thus, we only included the 20 items measuring anger expression.^[51]^

#### 2.2.7 World Health Organization Quality of Life scale abbreviated version

QOL was assessed using the Korean version of the WHOQOL-BREF,^[52,53]^ which defines QOL as an “individual's perception of their position in life in the context of the culture and value systems in which they live and in relation to their goals, expectations, standards, and concerns.”^[20,54]^ The WHOQOL-BREF addresses 4 domains (physical health, psychological health, social relationships, and environmental) as well as overall QOL and general health.^[20,53]^

#### 2.2.8 Buss-Perry aggression questionnaire

The Buss-Perry aggression questionnaire (AQ) is a 29-item instrument in which participants rate statements along a 5-point continuum from 1, which indicates “extremely uncharacteristic of me,” to 5, which indicates “extremely characteristic of me.” The AQ yields scores for 4 dimensions of aggression (physical aggression, verbal aggression, anger, and hostility).^[55,56]^ Higher scores on the AQ indicate higher levels of aggression.

#### 2.2.9 The short form of the Korean-Wechsler Adult Intelligence Scale

The K-WAIS SF^[57–59]^ was used to assess verbal and nonverbal intellectual functioning. The short form includes 4 subtests: vocabulary, arithmetic, block design, and picture arrangement.^[25]^

#### 2.2.10 Cambridge neuropsychological test automated battery

The Cambridge neuropsychological test automated battery (CANTAB) is a computerized neuropsychological assessment battery administered by computer with a touch-sensitive screen. It has been used neuropsychological research across age groups to study the development of a set of cognitive domains.^[60–62]^ Correlations between particular features of the CANTAB test scores and impairment in specific cognitive functions and psychiatric disorders have been demonstrated in very extensive data.^[62–71]^ The CANTAB tests used in this study included: the intraextra dimensional set shift (IED), a test of rule acquisition and reversal used to assess visual discrimination, attentional set-formation maintenance, and the ability to shift and flexibly allocate attention; the Stockings of Cambridge (SOC) test, used to assess spatial planning; the spatial span (SSP) test, which is a visuospatial analog of the Digit Span test used to assess working memory capacity; and the stop-signal test (SST), a classic stop-signal response-inhibition test used to assess the ability to inhibit a prepotent response.^[25]^

#### 2.2.11 Traditional neuropsychological test battery

The traditional neuropsychological test battery includes the following: the Korean Color-Word Stroop Test,^[72,73]^ which assesses sustained and selective attention, cognitive inhibition, and working memory; the trail making test (TMT),^[74,75]^ which assesses motor planning and cognitive shifting; and the verbal fluency (VF) test,^[76]^ which assesses cognitive fluency.^[25]^

### 2.3 Statistical analysis

We conducted exploratory data analyses prior to the formal analysis to identify and remove outliers to avoid the possibility of spurious results. The data analysis involved 3 main stages. First, independent *t* test was used to compare the IGD and HC groups with respect to age, gender, and education. A multivariate analysis of variance was used to compare the self-reported clinical data between the IGD and HC groups. A multivariate analysis of covariance (MANCOVA) was performed to compare the neuropsychological data (cognitive data) between the IGD and HC groups; because the IGD and HC groups differed significantly with respect to IQ, we set IQ score as a covariate in the MANCOVA analysis. The clinical and cognitive data were analyzed separately to minimize extraneous effects of the covariate. Second, pre- and post-treatment differences in the clinical and cognitive data were analyzed using paired *t* test. Third, stepwise multiple regression was performed to examine the associations between baseline clinical/cognitive data and changes in the IAT score (IAT score at pre-treatment minus IAT score at post-treatment), which allowed us to predict treatment prognosis for patients with IGD. Additionally, independent *t* test for baseline characteristics was performed to compare dropout IGD group (n = 25) with completed follow-up tests IGD group (n = 19). This process is needed because attrition bias might affect dropout rate of IGD group. All statistical analyses were performed using IBM SPSS Statistics ver. 21 (IBM Inc, Armonk, NY) and *P* values < 0.05 were considered significant.

## 3 Results

### 3.1 Demographic and clinical/cognitive data

The demographic and clinical/cognitive characteristics of participants are presented in Table 1. No differences were observed in age or education between the IGD and HC groups. The IGD group exhibited higher IAT (*P* < 0.001), BAI (*P* = 0.005), BDI (*P* < 0.001), PWI (*P* < 0.001), BIS-11 (*P* < 0.001), STAXI_trait anger (*P* = 0.02), and AQ (*P* = 0.001) scores than those of the HC group. In addition, the IGD group had lower QOL_physical health (*P* < 0.001), QOL_psychological health (*P* < 0.001), QOL_social relationships (*P* < 0.001), QOL_environmental (*P* = 0.02), and QOL_general health (*P* < 0.001) scores than those of the HC group. With regard to the cognitive data, the IGD group scored lower than the HC group with respect to the IQ (*P* = 0.009) and SST proportion of successful stops last sub-block (*P* = 0.002), indicating that the IGD group had lower inhibitory control ability than the HC group.

**Table 1:**
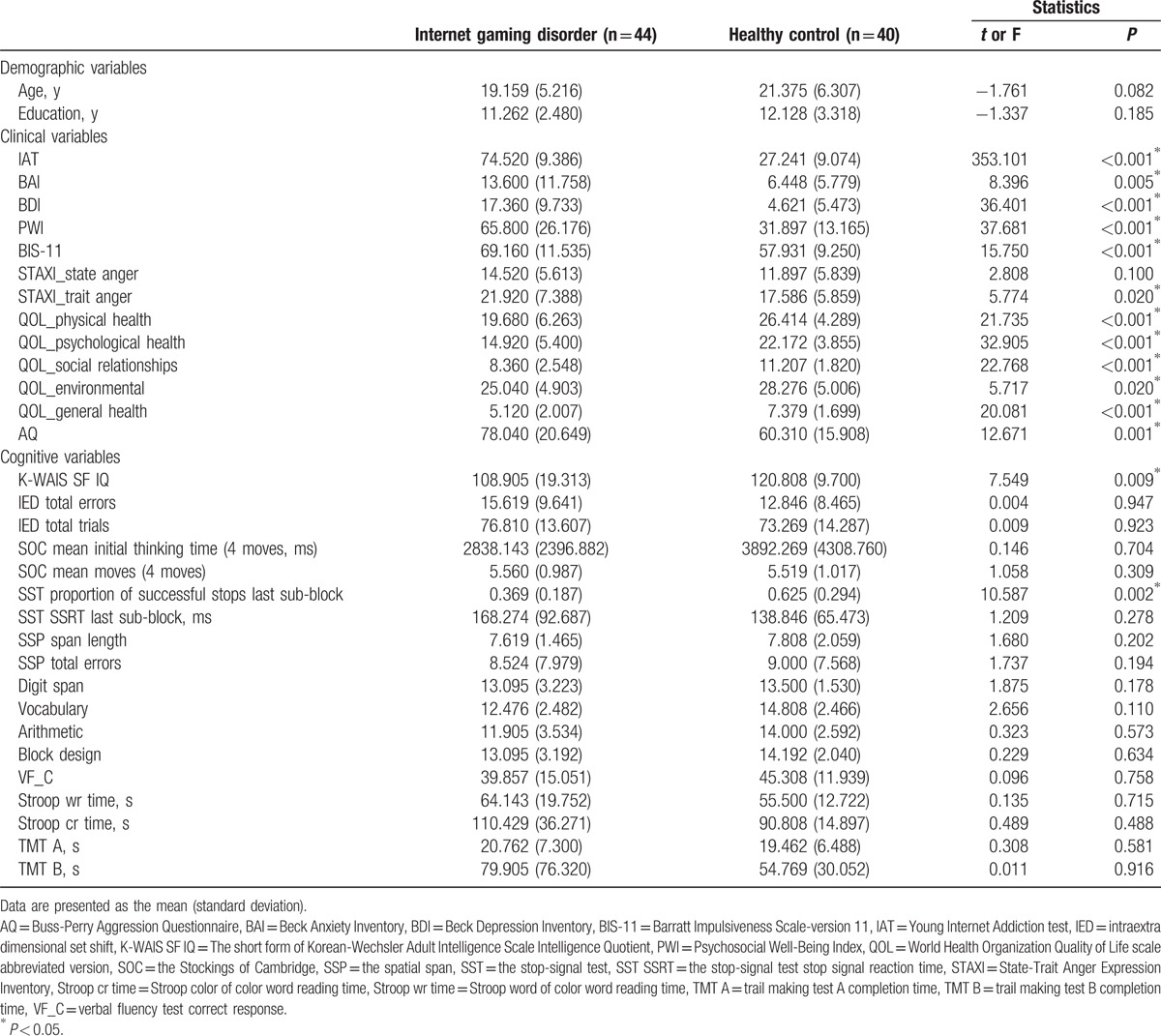
Baseline demographic, clinical, and cognitive characteristics of Internet gaming disorder and healthy control groups.

### 3.2 Clinical/cognitive changes after treatment

Table 2 shows the clinical and cognitive changes in the IGD group before and after 6 months of treatment. We found a significant decrease in the IAT (*P* = 0.003) and an increase in the QOL_psychological health (*P* = 0.03) scores. Completion times on the TMT B (*P* = 0.033) decreased and correct scores on the VF test increased (*P* = 0.019). Significant increases were observed for the scores on the SST proportion of successful stops last sub-block (*P* = 0.001) and SSP span length (*P* = 0.031), indicating that patients’ response inhibition and working memory were enhanced following treatment. In addition, a decrease in SOC mean moves (4 moves; *P* = 0.031) was detected, indicating that the spatial planning component of executive function improved after treatment.

**Table 2:**
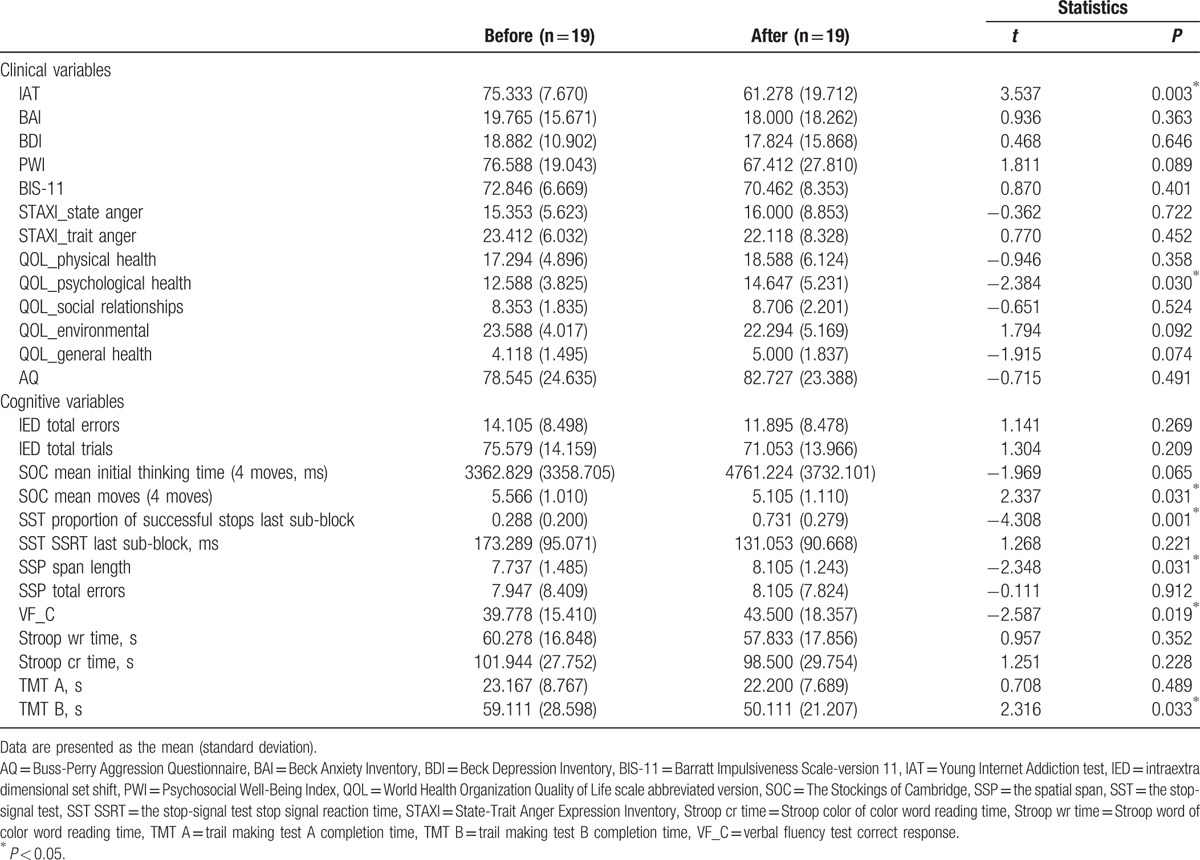
Clinical and cognitive changes in the Internet gaming disorder group after 6 months of outpatient management.

### 3.3 Predictive factors for improving IGD symptoms

The stepwise multiple regression analysis showed that the IAT scores were significantly predicted by the Digit Span scores and TMT B completion times at baseline (F = 8.624, df = 2, *P* = 0.008, *R*^2^ = 0.657, Table 3), indicating that the variables influencing the prognosis of IGD treatment were the Digit Span score (*t* = −4.144, *P* = 0.003) and TMT B time (*t* = −2.772, *P* = 0.022). Based on these results, we predicted a good prognosis for treatment of patients with IGD who had a low Digit Span score (β = −1.027) and a short TMT B completion time (β = −0.687) at baseline. No other variables were significant.

**Table 3:**
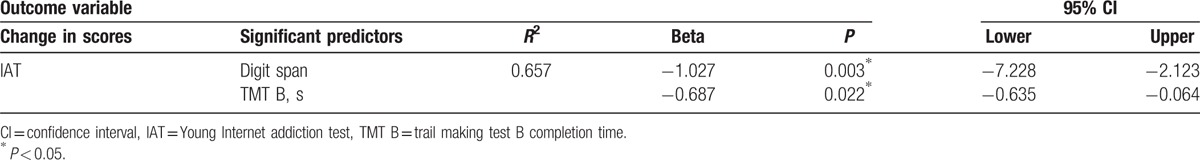
Significant results of the multiple regression analysis predicting the treatment response based on baseline cognitive scales and changes in the Internet addiction test scores.

We additionally administered independent *t* test in regard to baseline characteristics between dropout IGD group (n = 25) and completed follow-up IGD group (n = 19). Dropout IGD group showed higher QOL_psychological health (*P* = 0.011), QOL_general health (*P* = 0.008), SST proportion of successful stops last sub-block (*P* = 0.001) than those of completed follow-up IGD group. On the other hand, completed follow-up IGD group presented higher BAI (*P* = 0.008), PWI (*P* = 0.016), STAXI_trait anger (*P* = 0.020), and arithmetic (*P* = 0.010) than those of dropout IGD group. These results indicated that completed follow-up IGD group might have more mental health problems than dropout IGD group. But our study focused on completed follow-up IGD group to find out QOL and cognitive markers associated with longitudinal addiction symptom changes in IGD following outpatient management. We will discuss this issue in limitation section.

## 4 Discussion

This is the first study to investigate longitudinal changes in QOL and cognitive functioning, followed by an examination of the relationships of these changes with improved IGD symptoms after outpatient treatment. Furthermore, we offer predictions regarding IGD treatment prognosis via pre- and post-treatment IAT scores and baseline clinical/cognitive data.

Our baseline clinical and cognitive data showed significantly lower QOL and psychological well-being in the IGD group than in the HC group. In addition, the IGD group had more depression and anxiety symptoms than those in the HC group. The IGD group also exhibited impaired response inhibition relative to the HC group. This result is in agreement with previous SUD research, in which cocaine users had reduced response inhibition relative to HCs, as measured by the Go-No/Go task.^[31,77]^

The current findings indicate a significant decrease in addiction symptoms, as measured by the IAT score, and an increase in the QOL_psychological health domain. All patients with IGD who participated in this study had comorbid depressive or anxiety disorders. Additional research focusing on patients with IGD without comorbidities is needed to clarify the association between changes in QOL and mood states.

Moreover, neurocognitive tasks measuring executive functioning, particularly with regard to working memory and response inhibition, improved significantly after 6 months of outpatient treatment. Such cognitive changes have been demonstrated in previous studies of SUD. Within the first year of abstinence, performance on the TMT improves significantly in alcohol-dependent patients.^[78,79]^ In addition, many SUD studies have suggested that working memory function is linked to inhibitory control, such that reduced working memory function may facilitate drug cravings.^[31,80]^ These findings are relevant to the current results, which found that reduced inhibitory control in patients with IGD may reflect a greater desire for Internet gaming. Thus, the enhanced inhibitory control after treatment may be associated with diminished cravings for Internet gaming and improvements in IGD symptoms.

In addition, the present study found that low Digit Span scores and short completion times on the TMT B at baseline predicted a greater probability of improvement in IGD symptoms following treatment. The significant predictive factors pertain to working memory and executive functioning. In other words, patients with low working memory and high executive functioning at baseline are predicted to show a favorable response to IGD treatment.

Executive control can be explained as a unit of related but separable functions, including response inhibition, working memory, attention, problem solving, decision making, set shifting, and other functions.^[31,81]^ These functions have been operationalized by separate tasks and may serve as potential treatment targets for cognitive-enhancement approaches.^[31]^ Based on this background evidence, we infer that patients with IGD who have intact gross executive functioning but damage in the form of local lesions pertaining to working memory may show faster cognitive recovery than other patients with IGD. These findings need to be verified by neuroimaging research.

In the present study, patients with IGD exhibited significant improvements in their symptoms. We observed clinical/cognitive improvements with regard to subjective QOL, executive functioning, and response inhibition after 6 months of treatment. In particular, impaired inhibitory control in patients with IGD at baseline improved following treatment, indicating that impaired response inhibition may be an objective marker underlying the pathophysiology of IGD, which is consistent with a previous study.^[25]^

The present study has several limitations. First, sample size was small; therefore, the results may not be representative of all patients with IGD. In addition, only male participants were included in this study. Further study with a larger number of participants, including females, is needed to allow for broader generalization of the results. Second, the treatment modules were not well organized, consisting of the usual outpatient care, making it difficult to draw specific inferences about the effects of treatment. However, the main focus of this study was assessing subjective QOL and identifying neurocognitive markers associated with longitudinal symptom changes in IGD rather than on evaluating the effects of the treatment itself. In the future, it will be necessary to investigate the effects of specific treatment modules on QOL and neurocognitive markers in patients with IGD. Third, participants with IGD in the present study had comorbid depressive or anxiety symptoms, and these comorbidities may have influenced the effects of treatment. However, the patients showed no significant changes in the BDI or BAI scores after treatment, suggesting that improvements in addiction symptoms through treatment may be associated with increased psychological well-being, regardless of comorbid mood states. Also, most patients with IGD in clinical settings have various comorbidities; thus, the present findings may provide important clinical information regarding the underlying pathophysiology associated with IGD. Finally, high dropout rate of IGD group might lead to incomplete information and possibly flawed conclusions. But, it is well known that IGD correlated with impulsivity.^[23]^ The high dropout rate may be a feature of IGD, and, further, dropout subjects had less severe mental health problems than completed patients, indicating that completed patients were more likely to have motivations to the treatment.

Despite these limitations, our study adds to the existing evidence that IGD shares several features with SUD, including poor QOL and psychological well-being as well as impaired response inhibition. Furthermore, our study addressed longitudinal changes in QOL and cognitive functioning in patients with IGD following 6 months of outpatient management. Response inhibition may be an objective state marker in IGD, and the present results may be relevant to the establishment of treatment plans for patients with IGD.

## Acknowledgments

The authors would like to thank Ji Yoon Lee and Su Mi Park for helping to collect and analyze data.
